# Large Scale Population Assessment of Physical Activity Using Wrist Worn Accelerometers: The UK Biobank Study

**DOI:** 10.1371/journal.pone.0169649

**Published:** 2017-02-01

**Authors:** Aiden Doherty, Dan Jackson, Nils Hammerla, Thomas Plötz, Patrick Olivier, Malcolm H. Granat, Tom White, Vincent T. van Hees, Michael I. Trenell, Christoper G. Owen, Stephen J. Preece, Rob Gillions, Simon Sheard, Tim Peakman, Soren Brage, Nicholas J. Wareham

**Affiliations:** 1 Big Data Institute, Nuffield Department of Population Health, BHF Centre of Research Excellence, University of Oxford, Oxford, United Kingdom; 2 Institute of Biomedical Engineering, Department of Engineering Science, University of Oxford, Oxford, United Kingdom; 3 Open Lab, Newcastle University, Newcastle, United Kingdom; 4 School of Health Sciences, University of Salford, Manchester, United Kingdom; 5 MRC Epidemiology Unit, University of Cambridge, Cambridge, United Kingdom; 6 MoveLab, Institute of Cellular Medicine, Newcastle University, Newcastle, United Kingdom; 7 Population Health Research Institute, St George’s University of London, London, United Kingdom; 8 UK Biobank, Stockport, United Kingdom; Vanderbilt University, UNITED STATES

## Abstract

**Background:**

Physical activity has not been objectively measured in prospective cohorts with sufficiently large numbers to reliably detect associations with multiple health outcomes. Technological advances now make this possible. We describe the methods used to collect and analyse accelerometer measured physical activity in over 100,000 participants of the UK Biobank study, and report variation by age, sex, day, time of day, and season.

**Methods:**

Participants were approached by email to wear a wrist-worn accelerometer for seven days that was posted to them. Physical activity information was extracted from 100Hz raw triaxial acceleration data after calibration, removal of gravity and sensor noise, and identification of wear / non-wear episodes. We report age- and sex-specific wear-time compliance and accelerometer measured physical activity, overall and by hour-of-day, week-weekend day and season.

**Results:**

103,712 datasets were received (44.8% response), with a median wear-time of 6.9 days (IQR:6.5–7.0). 96,600 participants (93.3%) provided valid data for physical activity analyses. Vector magnitude, a proxy for overall physical activity, was 7.5% (2.35m*g*) lower per decade of age (Cohen’s d = 0.9). Women had a higher vector magnitude than men, apart from those aged 45-54yrs. There were major differences in vector magnitude by time of day (d = 0.66). Vector magnitude differences between week and weekend days (d = 0.12 for men, d = 0.09 for women) and between seasons (d = 0.27 for men, d = 0.15 for women) were small.

**Conclusions:**

It is feasible to collect and analyse objective physical activity data in large studies. The summary measure of overall physical activity is lower in older participants and age-related differences in activity are most prominent in the afternoon and evening. This work lays the foundation for studies of physical activity and its health consequences. Our summary variables are part of the UK Biobank dataset and can be used by researchers as exposures, confounding factors or outcome variables in future analyses.

## Introduction

Low physical activity is associated with an increased risk of morbidity and mortality [1]. However previous studies are predominantly based on self-reported participation in leisure time activity [2] from which it is difficult to quantify total physical activity across different domains [3]. This uncertainty makes it difficult to convert epidemiological association results into public health recommendations about the minimum level of physical activity required for health and the benefits of engaging in different durations of activity of different intensity. The development of objective methods for assessing physical activity has provided an opportunity to quantify the dose-response relationship of activity with health as a complement to the subjective assessment of self-reported participation in specific activities.

Accelerometry is the most widely used method for objective assessment of physical activity in population studies [4,5], and large studies from the UK [6–8], US [9], and Canada [10] indicate age gradients and differences between men and women; time-of-day and day-of-week differences in physical activity. Most earlier studies used accelerometers which were worn around the waist and during awake-time only, a protocol which can result in relatively large amounts of missing data [11]. Therefore, wrist-worn accelerometers are becoming more widely used as an objective measure of physical activity in cohorts in the UK [12], US [11], and Brazil [13]. These devices are water-proof and worn continuously day and night, resulting in higher levels of participant compliance [11,12]. Wrist-worn accelerometers have also been validated against established measures of physical activity energy expenditure [14,15].

Cohort studies which include hundreds of thousands of participants followed up over time are required in order to describe the relationship between physical activity and health outcomes that have a number of potential lifestyle, environmental, and genomic causes [16]. Objective assessment of physical activity in such large population-based cohorts has previously not been undertaken because of the challenges of cost and the feasibility of collecting, processing and analysing data on this large scale. In this paper we describe the methods used to collect and analyse physical activity by wrist-worn accelerometry in the UK Biobank cohort study and report the variation in activity in more than 100,000 participants by age, sex, and time.

## Methods

### Study Population

UK Biobank is a large prospective study with 500,000 participants aged 40–69 years when recruited in 2006–2010 [16]. The study has collected, and continues to collect, extensive phenotypic and genotypic detail about its participants, with ongoing longitudinal follow-up for a wide range of health-related outcomes. Only de-identified data are provided to researchers, who must sign a material transfer agreement, undertaking not to attempt to identify any participant, to keep the data secure, and to use it only for the purposes of the approved research [16]. Between February 2013 and December 2015, participants who had provided a valid email address were sent an email invitation to wear an accelerometer for seven days. The participant email addresses were chosen randomly, with the exception of the North West region which was excluded for much of the project due to participant burden concerns, as this area had been used to trial new projects. From June 2013, participants were sent devices in order of acceptance. This study was covered by the general ethical approval for UK Biobank studies from the NHS National Research Ethics Service on 17th June 2011 (Ref 11/NW/0382). None of the authors had direct contact with the study participants.

### Accelerometer & Data Collection

**Fig 1:**
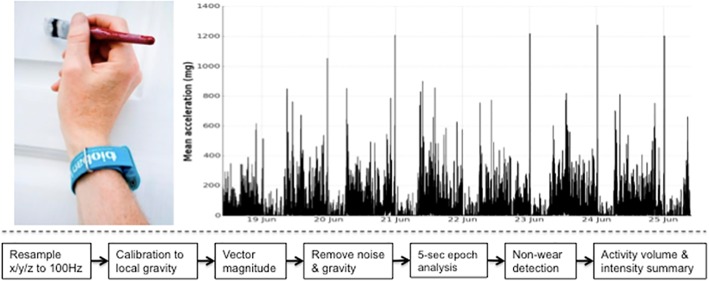
UK Biobank triaxial accelerometer and processing steps to extract physical activity information. Axivity AX3 triaxial accelerometer worn on dominant hand as used in UK Biobank (top left). Time series trace of processed accelerometer values after one week of wear (top right). Overview of process to extract proxy physical activity information from raw accelerometer data (bottom).

For objective assessment of physical activity, we used the Axivity AX3 wrist-worn triaxial accelerometer (see **Fig 1**), a commercial version of the Open Movement AX3 open source sensor (https://github.com/digitalinteraction/openmovement) designed by Open Lab, Newcastle University. This device demonstrated equivalent signal vector magnitude output on multi-axis shaking tests [17] to the GENEActiv accelerometer used in the Whitehall II [12], Fenland [15] and Pelotas cohorts [13]. The Axivity device facilitates transparent data processing analysis due to its open-source firmware platform and unforced sampling of raw measurement data. We set up the Axivity accelerometers to start at 10am two working days after postal dispatch, and capture triaxial acceleration data over a seven day period at 100Hz with a dynamic range of +-8*g*.

Participants were informed in the invitation email and device mail-out letter that the accelerometer should be worn continuously and that they should carry on with their normal activities. Participants were asked to start wearing the accelerometer immediately after receiving it in the post and to wear the monitor on their dominant wrist. They were also informed that the device was configured to automatically turn itself on soon after its arrival and off seven days later. Finally, participants were asked to mail the device back to the co-ordinating centre, in a pre-paid envelope, after the seven day monitoring period.

### Data Processing

To ensure different devices provided a similar output under similar conditions we calibrated the acceleration signals to local gravity using the procedure described by van Hees and colleagues [18]. Briefly, we identified stationary periods in ten second windows where all three axes had a standard deviation of less than 13.0 m*g*. These stationary periods were then used to optimise the gain and offset for each axis (9 parameters) to fit a unit gravity sphere using ordinary least squares linear regression. If insufficient data were available to conduct calibration for a given participant (where any of the three sensor axes did not have values outside a +- 300 m*g* range), we used the calibration coefficients from the previous (or if unavailable, the next) accelerometer record from the same device worn by a different participant. Clipped values, which occur when the sensor’s dynamic range of +-8*g* is exceeded, were flagged before and after calibration. Recording errors and ‘interrupts’, which could have occurred for example if participants tried to plug their accelerometer device into a computer, were also logged. Valid data were then resampled to 100 Hz using linear interpolation, except for interrupts lasting longer than 5 seconds which were set to missing. We calculated the sample level Euclidean norm of the acceleration in x/y/z axes, and removed machine noise using a fourth order Butterworth low pass filter with a cutoff frequency of 20Hz. In order to separate out the activity-related component of the acceleration signal, we removed one gravitational unit from the vector magnitude, with remaining negative values truncated to zero [12,13].

To describe the overall level and distribution of physical activity intensity, we combined the sample level data into five second epochs for summary data analysis, maintaining the average vector magnitude value over the epoch. To represent the distribution of time spent by an individual in different levels of physical activity intensity, we generated an empirical cumulative distribution function from all available five second epochs [13,19]. We removed non-wear time, defined as consecutive stationary episodes lasting for at least 60 minutes where all three axes had a standard deviation of less than 13.0 m*g* [12,14]. We imputed non-wear data segments using the average of similar time-of-day vector magnitude and intensity distribution data points with one minute granularity on different days of the measurement, as in previous studies [12,14]. This imputation accounts for potential wear time diurnal bias where, for example, if the device was systematically less worn during sleep in an individual, the crude average vector magnitude during wear time would be a biased overestimate of the true average. We then constructed a physical activity outcome variable by averaging all worn and imputed values. Our analysis is freely available and hosted as an open source software project at https://github.com/activityMonitoring/biobankAccelerometerAnalysis

### Data Analysis

For process evaluation we generated descriptive statistics on the number of participants and devices used. We recorded the number of participants who had insufficient data for calibration. We also noted the percentage of data recording errors caused by interrupts and clipped values, both before and after calibration. Furthermore, we described the number of participants who provided different amounts of wear time. We then excluded individuals with less than three days (72 hours) of wear data or who did not have wear data in each one-hour period of the 24-hour cycle. We defined these criteria after finding 72 hours of wear were needed to be within 10% of a complete seven day measure (using intraclass correlation coefficients) in missing data simulations on 29,765 participants who had perfect wear time compliance (see S1 Fig).

Descriptive statistics were used to report device wear time compliance in hours and accelerometer measured physical activity in milli-gravity units (m*g*). Age groups were categorised into decade bands from ages 45–79 years. Age and seasonal (with Spring starting on 1^st^ March) differences in device wear-time were examined using the Kruskal-Wallis test, while sex differences were examined using the Wilcoxon-Mann Whitney test. Differences in wear-time distribution were examined using the Friedman test for time-of-day (six hour quadrants, e.g. 00:00–05:59, 06:00–11:59, etc.) and Wilcoxon signed ranks test for days (weekdays versus weekend days), within individuals for men and women separately. Mean acceleration vector magnitude differences by age group were investigated using one-way repeated measures ANOVA for time-of-day (six hour quadrants) and days (weekdays versus weekend days), within individuals for men and women separately. Seasonal differences in mean acceleration vector magnitude were investigated using two-way ANOVA between age groups, for men and women separately. We used R to perform all statistical analyses [20]. Given the size of this dataset, almost all of our findings show robust statistical significance (p<0.001). We therefore do not report such small p-values. Box plots were used to show differences between groups in this cross-sectional data similar to the approach taken previously [8,13,21].

## Results

**Fig 2:**
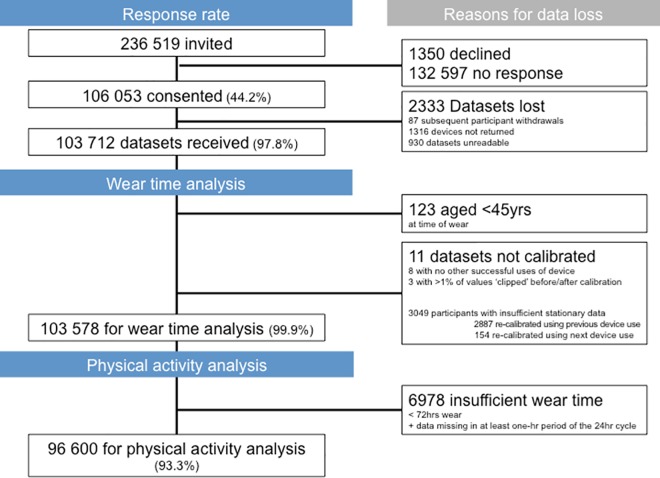
Participant flow chart; the UK Biobank study 2013–2015 (n = 103,712).

A total of 236,519 UK Biobank participants were approached, of whom 106,053 agreed to wear a physical activity monitor (44.8%). The median time between each participant being invited to take part and being sent a device was 113 days (IQR: 73–137 days). Fig 2 shows that 103,712 datasets were received for data analysis. 123 participants were excluded as they were aged less than 45 years. Eleven participants were excluded from further analysis; eight because the calibration by the preceding or subsequent measurement was not possible due to insufficient data; and three participants due to unreliable device data. A total of 4043 devices were used on a median number of 27 occasions (IQR: 8–39). The median time between each device being posted was 17.0 days (IQR: 15.8–19.8) with a median of 832 devices (IQR: 629–994) posted each week.

Calibration of the data to local gravity greatly reduced the error in the assessment of acceleration with the root mean square error of stationary points falling from an average of 81.8 m*g* (95% CI: 81.6–82.1) to an average of 2.6 m*g* (95% CI: 2.6–2.6). However, 2.9% (n = 3049) of participants had insufficient stationary data to inform the calibration. These individual records were calibrated using stationary episodes from the previous (n = 2887) or next (n = 154) use of the same device by different participants. The influence of clips (readings beyond the sensor’s dynamic range of +-8*g*) before (median: 160, IQR: 62–393) and after (median: 169, IQR: 67–410) calibration, interrupts (median: 0, IQR: 0–0), and errors such as clips or missing readings (median: 200, IQR: 66–355) was negligible, with respect to the median of 58.6 million data readings (IQR: 56.0–60.1 million).

**Fig 3:**
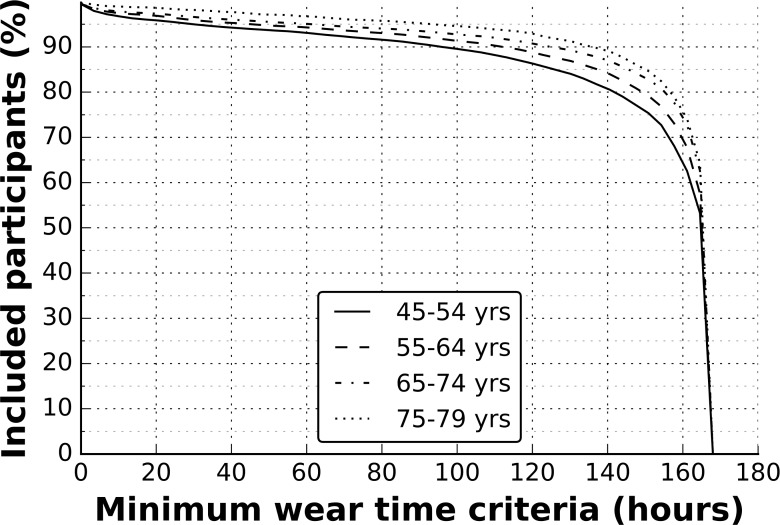
Cumulative distribution function of accelerometer wear time compliance; the UK Biobank study 2013–2015 (n = 103,578).

Fig 3 illustrates that 80.6% of participants wore the device for at least 150 hours out of a scheduled 168 hours. Men wore the device for a median of 166.3 hours (IQR: 157.7–168.0) and were slightly more compliant than women who wore the device for a median of 165.6 hours (IQR: 156.7–167.0). Table 1 shows that older age groups had marginally higher levels of compliance than younger age groups. Analysis of wear time compliance by age on a linear scale shows that on average there was a difference of 2 hours 18 minutes (1.6%) for each decade. In addition, Table 1 indicates minimal differences in the wear time compliance by time-of-day and week-weekend day. No wear-time differences were found by season. We removed 6978 (6.7%) participants who had insufficient wear data for our remaining analyses on accelerometer measured physical activity.

**Table 1:** Wear-time compliance and acceleration vector magnitude by age, day, time of day, and season, stratified by sex: The UK Biobank study 2013–2015 (n = 103,578).

	Wear time [median (IQR) hours]		Acceleration vector magnitude [mean +- stdev m*g*]	
	Women	Men	Women	Men
**Age (yrs)** ^A^				
45–54	164.9 (152.4–167.0)	165.4 (149.5–168.0)	31.2 +- 8.7	31.1 +- 9.7
	_(n = 12,586)_	_(n = 8655)_	_(n = 11,572)_	_(n = 7838)_
55–64	165.4 (156.0–167.0)	165.8 (156.5–168.0)	29.1 +- 8.0	28.8 +- 8.8
	_(n = 21,322)_	_(n = 14,410)_	_(n = 19,890)_	_(n = 13,362)_
65–74	165.6 (159.1–168.0)	166.8 (160.8–168.0)	26.6 +- 7.1	25.6 +- 7.7
	_(n = 22,821)_	_(n = 20,595)_	_(n = 21,489)_	_(n = 19,385)_
75–79	165.6 (158.9–167.0)	166.8 (162.6–168.0)	23.9 +- 6.5	22.9 +- 6.8
	_(n = 1494)_	_(n = 1695)_	_(n = 1436)_	_(n = 1628)_
p value	p<0.001	p<0.001	p<0.001	p<0.001
**Time of day** ^B^				
0–5.59 _am_	40.9 (36.0–42.0)	42.0 (36.6–42.0)	4.4 +- 3.1	4.9 +- 4.4
	_(n = 58,223)_	_(n = 45,355)_	_(n = 54,387)_	_(n = 42,213)_
6–11.59 _am_	41.0 (38.9–42.0)	42.0 (39.0–42.0)	38.6 +- 14.9	37.4 +- 16.4
	_(n = 58,223)_	_(n = 45,355)_	_(n = 54,387)_	_(n = 42,213)_
12–5.59 _pm_	42.0 (40.3–42.0)	42.0 (40.3–42.0)	44.3 +- 13.8	42.9 +- 16.0
	_(n = 58,223)_	_(n = 45,355)_	_(n = 54,387)_	_(n = 42,213)_
6–11.59 _pm_	42.0 (39.5–42.0)	42.0 (40.2–42.0)	26.4 +- 10.4	24.9 +- 11.5
	_(n = 58,223)_	_(n = 45,355)_	_(n = 54,387)_	_(n = 42,213)_
p value	p<0.001	p<0.001	p<0.001	p<0.001
**Day** ^C^				
Weekday	23.7 (22.5–24.0)	23.8 (22.6–24.0)	28.5 +- 8.2	27.5 +- 9.0
	_(n = 58,223)_	_(n = 45,355)_	_(n = 54,387)_	_(n = 42,213)_
Weekend	24.0 (22.9–24.0)	24.0 (23.3–24.0)	28.0 +- 9.4	27.1 +- 10.8
	_(n = 58,223)_	_(n = 45,355)_	_(n = 54,387)_	_(n = 42,213)_
p value	p<0.001	p<0.001	p<0.001	p<0.001
**Season** ^D^				
Spring	165.6 (156.2–167.5)	166.1 (157.4–168.0)	28.8 +- 8.0	28.1 +- 9.1
	_(n = 13,365)_	_(n = 10,224)_	_(n = 12,480)_	_(n = 9,469)_
Summer	165.4 (156.2–168.0)	166.3 (157.4–168.0)	28.8 +- 8.1	28.2 +- 8.7
	_(n = 15,450)_	_(n = 11,943)_	_(n = 14,353)_	_(n = 11,016)_
Autumn	165.6 (157.2–167.0)	166.3 (158.2–168.0)	28.3 +- 8.0	27.3 +- 8.7
	_(n = 17,213)_	_(n = 13,506)_	_(n = 16,157)_	_(n = 12,633)_
Winter	165.6 (156.7–168.0)	166.3 (157.9–168.0)	27.7 +- 7.8	26.3 +- 8.4
	_(n = 12,195)_	_(n = 9,682)_	_(n = 11,397)_	_(n = 9,095)_
p value	p = 0.289	p = 0.104	p<0.001	p<0.001

^*A*^ *Age*: Kruskal-Wallis test used to compare wear-time distributions, and one-way analysis of variance test used to compare acceleration vector magnitude means. Sum wear time hours for week displayed (max = 168.0).

^*B*^ *Time of day*: Friedman test used to compare wear-time distributions within individuals, and repeated one-way analysis of variance test used to compare acceleration vector magnitude means within individuals and between age groups. Sum wear time hours for time quadrant over a week displayed (max = 168.0).

^*C*^ *Day*: Wilcoxon test used to compare wear-time distributions within individuals, and repeated one-way analysis of variance test used to compare acceleration vector magnitude means within individuals and between age groups. Average wear time hours for day displayed (max = 24.0).

^*D*^ *Season (Spring starting on 1*^*st*^ *March)*: Kruskal-Wallis test used to compare wear-time distributions, and two-way analysis of variance test used to compare acceleration vector magnitude means between age groups. Sum wear time hours for week displayed (max = 168.0).

**Fig 4:**
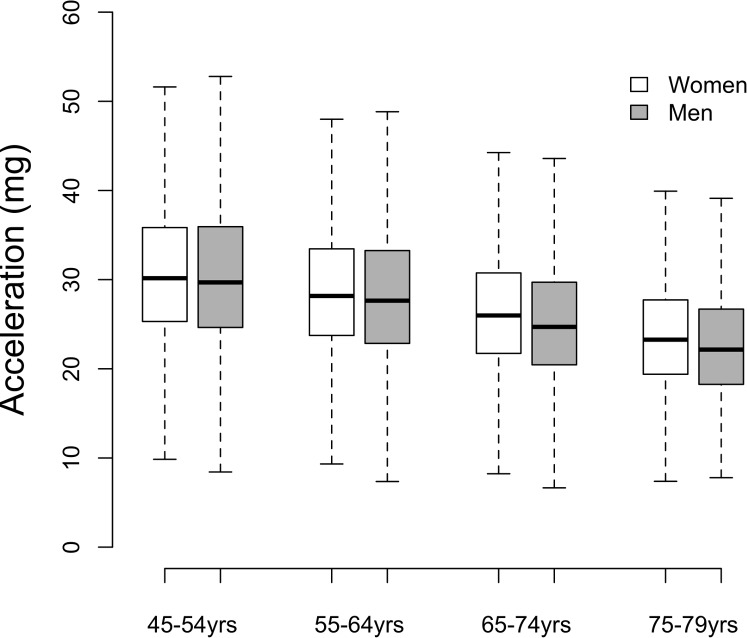
Acceleration vector magnitude by sex and age; the UK Biobank study 2013–2015 (n = 96,600).

Table 1 describes the variation in mean vector magnitude, the summary measure of accelerometer measured physical activity, by age and sex in the sub-group of 96,600 participants who had good wear time compliance. Vector magnitude was higher in women than men, apart from those aged 45–54 years (p = 0.98). The mean effect size for these sex differences was small (0.09), ranging from 0.01 for 45–54 years to 0.15 for 75–79 years. There was strong evidence of accelerometer measured physical activity differing by age group in both men and women. The mean physical activity in the age group 45–54 years was 31.17 m*g* (SD 9.10) and was, on average 7.5% or 2.35 m*g* lower per decade. The mean effect size for these age differences was large, at 0.89 for women and 0.9 for men. Fig 4 shows the distribution of the data within age and sex strata, highlighting that although there appears to be an overall decline in average physical activity with increasing age, there is considerable overlap in the distributions with many older participants being more active than those in the youngest age category.

**Fig 5:**
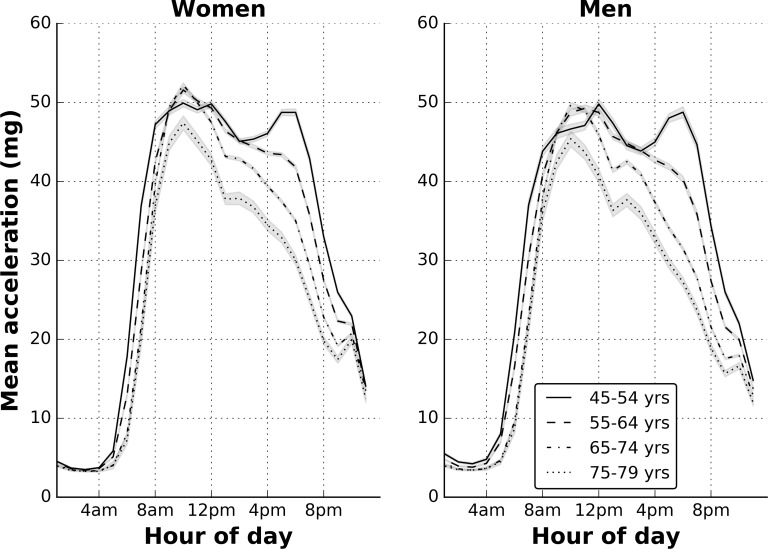
Variation in mean acceleration across the day by age and sex: the UK Biobank study 2013–2015 (n = 96,600). Shading bounds represent two standard errors.

**Fig 6:**
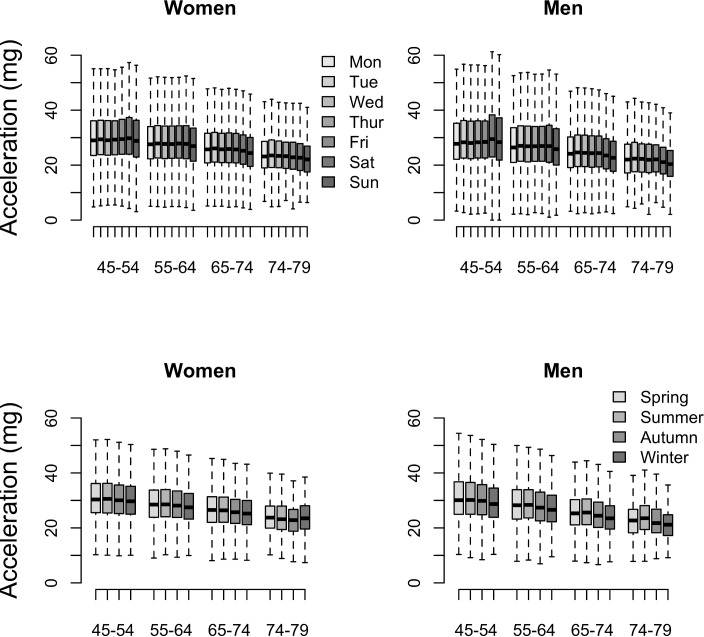
Acceleration vector magnitude by day of the week (top), season (bottom), age, and sex: the UK Biobank study 2013–2015 (n = 96,600).

Fig 5 shows the mean physical activity level by hour of day averaged across the whole measurement period by age and sex. It shows that the effect size for physical activity differences between age groups are most apparent in the afternoon (0.74 for women and 0.69 for men) and evening (1.06 for women, 1.12 for men) with smaller differences by age group in the morning (0.56 for women, 0.46 for men). Weekdays and weekend days differed, with vector magnitude higher at weekdays except for those aged 45–54 years. However, the mean effect size for these day differences was small (0.10), ranging from 0.04 to 0.15 across female age groups and 0.11 to 0.18 for male age groups (see Fig 6). Seasonality also differed, with vector magnitude lower during winter months except for women aged 75–79. However, the mean effect size for these season differences was small (0.21), ranging from 0.09 to 0.18 across age groups in women and 0.17 to 0.41 across age groups in men (see Fig 6).

**Fig 7:**
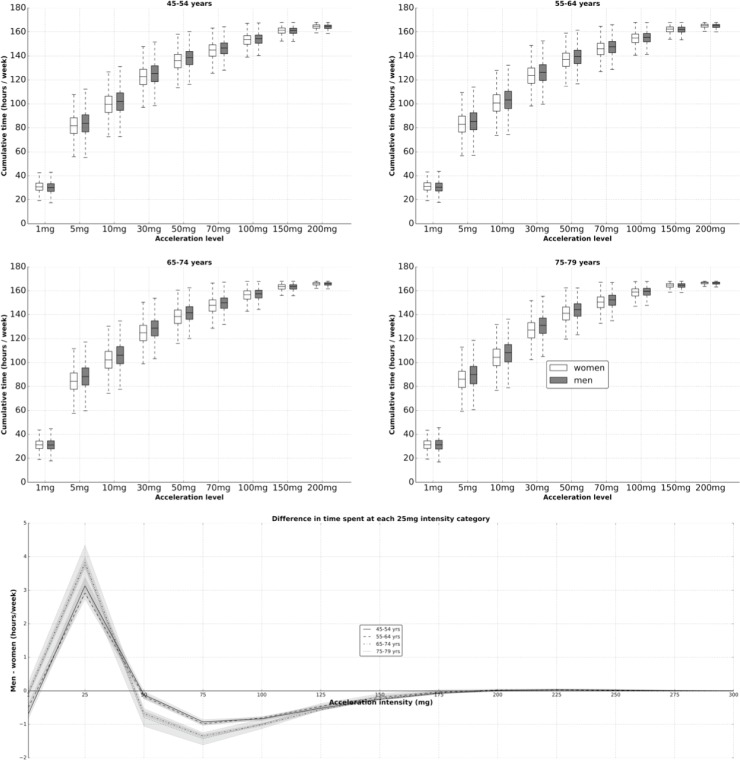
Cumulative time spent in various acceleration categories by sex and age (top), and sex differences by age and intensity level (bottom); the UK Biobank study 2013–2015 (n = 96,600).

To illustrate time spent at different physical activity intensities, Fig 7 plots the empirical cumulative distribution function of the five second sample values for each subgroup. The bottom part of this figure shows sex differences in the distribution of physical activity intensity, for each age group. For example, men spend more time at or below 25 m*g* than women (122.6 versus 119.3 hours), but also slightly more time above 225 m*g* than women (2.18 versus 2.09 hours).

## Discussion

Developments in the technology supporting objective assessment of physical activity have now made it possible to consider assessing this behaviour objectively in large scale population-based cohort studies as an adjunct to more traditional assessment of self-reported participation in activities within different domains of life. However, even with those technological developments, it has previously been unknown whether it would be possible for this approach to be acceptable to participants and whether it would prove to be feasible to collect, analyse and interpret data from over one hundred thousand participants. This report from the UK Biobank study shows that 45% of participants who were invited to wear a monitor accepted the invitation. It also shows that measuring activity with a wrist worn device is highly acceptable to participants as manifest by the very high proportion of people in whom the data were of high quality and completeness. By necessity in the UK Biobank Study participants were invited to wear the monitor some time after recruitment to the baseline visit. As with all add-on measurements that are conducted on a different occasion, there will be participants who do not accept the invitation to participate. Other studies in which wrist worn accelerometers are part of the protocol for a baseline visit, rather than a separate add-on, will be likely to achieve higher participation levels.

We have shown that mean vector magnitude in this population was greater in women than men, apart from those aged 45–54 years. Our findings also suggest that men spend more time than women in what might be considered low or sedentary levels of physical activity, while women spend more time in moderate levels of activity. Whether these results indicate true differences in physical activity between sexes or are a function of a between-sex difference in the relationship between wrist acceleration and true activity remains to be investigated. For example, a recent study of 1695 UK men and women reported physical activity energy expenditure to be 7% lower in women compared to men for the same level of non-dominant wrist acceleration[15]. With respect to age, we observed a marked overall difference in the summary measure of physical activity by age, with older participants having levels of activity that are, on average, 7.5% lower for each 10 year age difference. These differences by age group are similar to other population-based studies [6,9] that have used hip worn accelerometers. With respect to time, older participants are much less active than younger participants during afternoons/evenings than in the morning, which mirrors previous findings in older UK adults using hip worn accelerometers [7]. There were small differences between weekday and weekend day physical activity, and also small seasonal differences in activity. We have not generalised the overall descriptive findings to the UK population since the UK Biobank was established as an aetiological study rather than one aimed at population surveillance [6,9].

We have extracted objective physical activity information from 103,578 participants aged 45–79, who were asked to wear accelerometers for seven days on their dominant wrist. The strengths of this study include its use of objective measures of physical activity, excellent participant compliance, unprecedented scale, and use of reproducible methods. For example, >93% of participants provided more than 72 hours of wear time with no missing data bias by time of day. The overall levels of participant compliance in the UK Biobank mirror findings in other studies that have used wrist-worn accelerometers in thousands of participants [11–13]. Wrist-worn accelerometers are not only highly acceptable to participants, but are also valid measures of physical activity energy expenditure. A recent free-living study reported that wrist acceleration explained 44% of the variance in physical activity energy expenditure estimated from individually calibrated combined heart rate and movement sensing[15]. Laboratory-based studies have demonstrated that the signal from wrist-worn devices correlates with physical activity energy expenditure as well as traditional waist-worn devices (left wrist R = 0.86; right wrist R = 0.83; waist R = 0.87) [22]. Thus the relative validity between wrist-worn devices is similar and the association of accelerometer measured physical activity with health outcomes within a study is not dependent upon which wrist was chosen. However, the comparison of absolute values between studies would need to be mindful of which wrist was selected for individual studies. More robust validation studies of physical activity information from raw wrist-worn accelerometer data are needed to enhance the interpretation of this signal.

Even though we used relatively simple summary measures in these analyses, their derivation still involved several critical data processing decisions, the alteration of which would have large effects on the derived physical activity variables [18]. For example, there is uncertainty on how to address negative values during the gravity removal process. Furthermore, it is not possible to perfectly separate static and dynamic acceleration (for example gravity and physical activity) from the measurement of triaxial acceleration alone. Therefore, we produced summary statistics of vector magnitude which do not attempt this separation. In addition, absolute and truncated Euclidian norm minus one and high-pass filtered vector magnitude (all of which attempt to separate activity from gravity) were generated too. We found that while the magnitude of these variables changes, their correlation was very strong (>0.95), which provides confidence in our chosen metric for association studies. Uncertainty also exists on the best method to identify non-wear episodes, and the size of epoch on which to base distributions of physical activity intensity. While the derived factors are only the most basic variables that can be extracted from the raw 100Hz triaxial acceleration data, future projects will be able to build on this foundation to derive additional parameters describing other aspects of physical activity, sedentary behaviour and sleep.

In conclusion, the collection and processing of this large accelerometer dataset in a prospective cohort study lays the foundation for studies of physical activity and its health consequences. The summary variables that we have constructed are now part of the UK Biobank dataset and can be used by researchers as exposures, confounding factors or outcome variables in future analyses.

## Supporting Information

S1 Fig Minimum wear time criterion. One challenge is to determine the minimum amount of time participants should wear an accelerometer to get a reliable measure of their physical activity status. Therefore, using 29 765 participants who had complete wear time compliance, we simulated the effect of only having 24–168 hours of data (1–7 days). Using intraclass correlation coefficients, at least 72 hours (3 days) of wear were needed to be within 10% of the true stable seven day measure.
(DOCX)
