# Supplementary material for: Large Scale Population Assessment of Physical Activity Using Wrist Worn Accelerometers: The UK Biobank Study
Source: PLoS One. 2017 Feb 1;12(2):e0169649. doi: 10.1371/journal.pone.0169649 (PMC5287488; doi:10.1371/journal.pone.0169649)

**SUPPLEMENT 1 – Minimum wear time criterion**

One challenge is to determine the minimum amount of time participants should wear an accelerometer to get a reliable measure of their physical activity status. Therefore, using 29 765 participants who had complete wear time compliance, we simulated the effect of only having 24 - 168 hours of data (1-7 days). Using intraclass correlation coefficients, at least 72 hours (3 days) of wear were needed to be within 10% of the true stable seven day measure.


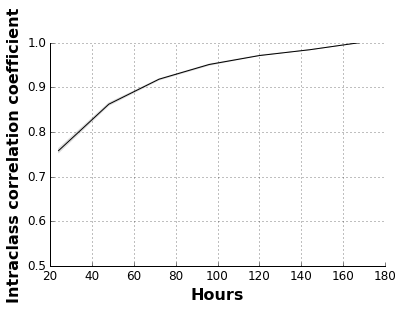

Supplement: S1 Fig — One challenge is to determine the minimum amount of time participants should wear an accelerometer to get a reliable measure of their physical activity status. Therefore, using 29 765 participants who had complete wear time compliance, we simulated the effect of only having 24–168 hours of data (1–7 days). Using intraclass correlation coefficients, at least 72 hours (3 days) of wear were needed to be within 10% of the true stable seven day measure. (DOCX) [file pone.0169649.s001.docx]
